# The Association Between Periconceptional Consumption of Ultra-Processed Food and the Incidence of Adverse Pregnancy Outcomes

**DOI:** 10.3390/nu18040627

**Published:** 2026-02-14

**Authors:** Raven Hall, Alyssa M. Hernandez, Suzette Rosas-Rogers, Melodee Liegl, Amy Y. Pan, Catherine Cohen, Anna Palatnik

**Affiliations:** 1 Department of Obstetrics and Gynecology, Medical College of Wisconsin, Milwaukee, WI 53226, USA; alyhernandez@mcw.edu (A.M.H.);; 2 Bioinformatics and Quantitative Child Health, Medical College of Wisconsin, Milwaukee, WI 53226, USA; mliegl@mcw.edu (M.L.);; 3 Colorado School of Public Health, University of Colorado Anschutz Medical Campus, Aurora, CO 80045, USA; catherine.cohen@cuanschutz.edu; 4 Cardiovascular Center, Medical College of Wisconsin, Milwaukee, WI 53226, USA

**Keywords:** pregnancy, maternal diet, ultra-processed food, preterm birth, preeclampsia

## Abstract

**Background/Objectives**: Increasing popularity, convenience, and access to processed foods are shifting the composition of dietary intake from whole to ultra-processed foods (UPF). This study aimed to assess the association between periconceptional UPF consumption and the incidence of adverse pregnancy outcomes (APOs). **Methods**: This was a secondary analysis of the Nulliparous Pregnancy Outcomes Study: Monitoring Mothers-to-Be (nuMoM2b). Patients were excluded if they were missing periconceptional diet data or if their pregnancy ended before 20 weeks. Food Frequency Questionnaire items were categorized using the NOVA Scale to calculate the proportion of total energy intake comprised of UPF (% kcal/day). Bivariate and multivariate analyses examined the relationships between UPF intake and preterm birth, hypertensive disorders of pregnancy (HDP), gestational diabetes (GDM), small-for-gestational-age (SGA) infants, large-for-gestational-age (LGA) infants, and fetal or neonatal demise. **Results**: A total of 6693 participants were included in the analysis. The sample was predominantly White (78%) and not Hispanic (84%), and a majority of participants had commercial insurance (76%). UPF accounted for an average of 51.3 ± 12.7% of participants’ daily total energy intake. Mean UPF intake was higher among patients who identified as Black or non-Hispanic, patients with public insurance, less than a high school education, a household income below the federal poverty level (all *p*-values < 0.001), patients with chronic hypertension (*p* = 0.02), and patients who delivered vaginally (*p* = 0.002). Patients with preterm birth, HDP, SGA infants, and fetal or neonatal demise all had significantly higher proportions of daily UPF intake compared to patients without these adverse outcomes. After adjusting for potential confounders, higher UPF intake remained significantly associated with preterm birth (AOR 1.11, 95% CI 1.02–1.21) and HDP (AOR 1.05, 95% CI 1.001–1.11). **Conclusions**: On average, more than half of participants’ daily energy intake was from UPF, and higher UPF intake correlated with several adverse pregnancy outcomes. Future efforts should focus on improving nutritional literacy regarding UPF consumption in pregnancy.

## 1. Introduction

Dietary habits during pregnancy are modifiable health behaviors that may impact birth outcomes as nutrients play an important role in pregnancy and ensuring sufficient fuel transfer for the growing fetus [1,2,3,4]. Current clinical nutrition recommendations for pregnant women encourage a balanced diet of fruits, vegetables, proteins, and whole grains, while limiting consumption of foods high in sugar, saturated fat, and salt [5]. However, the vast majority of pregnant women do not adhere to the Dietary Guidelines for Americans [6,7], partly due to generally low levels of nutritional literacy among pregnant populations [8], as well as limited access to affordable, nutritious food [9].

Studies show that around 50% of pregnant women’s daily diet consists of ultra-processed foods (UPF) [10,11], defined as foods that are highly altered to increase palatability and shelf-life. They often contain additives rarely found in kitchens, such as emulsifiers, chemical sweeteners, and hydrogenated fats [12]. Recent studies have linked UPF consumption to increased obesity, type 2 diabetes, and cardiovascular disease risk in nonpregnant populations [13,14,15]. Moreover, an observational study including nearly 45,000 participants with seven years of follow-up demonstrated a significant association between UPF consumption and all-cause mortality after adjusting for several potential confounders [16].

The literature examining the association between UPF consumption and pregnancy outcomes is growing. While a few observational studies have demonstrated associations between UPF intake and maternal–fetal outcomes, these studies have been limited by sample size, warranting replication in other populations [17,18,19]. Further research is needed to clarify the relationship between maternal consumption of UPFs and adverse pregnancy outcomes, such as preterm birth and preeclampsia, which are major drivers of maternal and neonatal morbidity, and continue to rise in the United States [20,21,22,23]. Therefore, we performed a secondary analysis of a large prospective observational cohort study of pregnant women to evaluate the association between maternal UPF intake and several adverse pregnancy outcomes.

## 2. Materials and Methods

This was a secondary analysis of the Nulliparous Pregnancy Outcomes Study: Monitoring Mothers-to-Be (nuMoM2b), a prospective observational cohort study conducted from 2010 to 2013 [24]. All 10,037 participants were first-time mothers ≥13 years old with singleton pregnancies who enrolled between 6 and 14 weeks’ gestation. Patients were excluded from participating in nuMoM2b if they had a history of recurrent pregnancy loss, lethal fetal malformation, fetal aneuploidy, surrogate pregnancy, or planned pregnancy termination. As part of the nuMoM2b study, participants were asked to consent to sharing their data after study termination. The nuMoM2b dataset was accessed from the Eunice Kennedy Shriver National Institute of Child Health and Human Development Data and Specimen Hub (DASH), and the study was registered with our Institutional Review Board (PRO00051869, May 2024).

For the current analysis, all nuMoM2b participants were included if they had available food frequency data. Participants were excluded if they had a spontaneous or iatrogenic abortion before 20 weeks’ gestation. In nuMoM2b, periconceptional dietary data were collected using the Modified Block 2005 Food Frequency Questionnaire (FFQ) prior to 14 weeks’ gestation. The FFQ is a validated instrument that asks about 120 food and beverage items and was used to assess maternal diet in the three months prior to conception [25]. We categorized all FFQ items using the NOVA scale, which was validated to group foods by their level of industrial, mechanical, and physical processing, with UPFs falling into group 4 [26]. We then estimated the average caloric density of each line item in the FFQ, categorized as NOVA group 4, using the FoodData Central (FDC) published by the U.S. Department of Agriculture [27]. For foods with multiple brands or preparation options indexed in the FDC, we averaged the caloric density across up to 13 examples of the FFQ item. We then calculated the total daily intake of group 4 foods by multiplying the average caloric density by the grams of daily intake reported in the FFQ. Total group 4 food intake was divided by daily total energy intake (TEI) to determine the percentage of daily kilocalories (% kcal/day) comprised of UPFs. We further characterized the average intake of UPFs by food group to depict daily dietary patterns of UPF consumption.

We calculated summary statistics for demographics, baseline clinical characteristics, and adverse pregnancy outcomes, including preterm birth (delivery prior to 37 weeks), small-for-gestational-age birthweight infants (SGA, birthweight < 10th percentile [28,29]), large-for-gestational-age birthweight infants (LGA, birthweight > 90th percentile), hypertensive disorders of pregnancy (HDP, including gestational hypertension, preeclampsia, superimposed preeclampsia, or eclampsia per the American College of Obstetricians and Gynecologists diagnostic criteria [30]), gestational diabetes (GDM, diagnosed by using a two-step screening approach [31]), and offspring morbidity (defined as fetal demise after 20 weeks’ gestation or neonatal demise within 28 days of life). UPF intake was modeled as a continuous variable rather than categorized into quantiles. This approach preserves information and statistical power and avoids known limitations of categorizing continuous exposures [32,33,34,35,36,37]. Linearity between UPF intake and the log-odds scale of each adverse pregnancy outcome was assessed and supported, with no evidence of threshold or non-linear effects. In the absence of a clinically established cutoff, continuous modeling was considered the most appropriate analytic approach.

Data were reported as mean ± standard deviation (SD) or median and interquartile range (IQR) as appropriate. Two-sample *t*-tests were used to compare mean UPF intake across maternal demographic and baseline clinical characteristics. Spearman correlation coefficients (*r_s_*) were used to test correlations between continuous baseline clinical characteristics and UPF intake. Two-sample *t*-tests were used to compare mean UPF intake between groups for all adverse pregnancy outcomes after confirming homogeneity of variances. Finally, we performed univariate and multivariate logistic regressions to examine the independent associations between increasing UPF intake (calculated per ten percent changes in UPF intake) and adverse pregnancy outcomes. Odds ratios (ORs) and 95% confidence intervals (CIs) were generated for each ten percent increase in UPF intake. Adjusted odds ratios were generated in the same manner after controlling for potential confounders identified during the bivariate analysis. A *p*-value < 0.05 was considered statistically significant. SAS Version 9.4 (SAS Institute Inc., Cary, NC, USA) and IBM SPSS Statistics Version 29.0 were used for data analysis.

## 3. Results

After excluding individuals with missing FFQ data, 7435 participants were eligible for inclusion in this secondary analysis. Furthermore, due to significant outliers in TEI, we excluded any participants with TEI falling beyond the <5th or >95th percentiles. This resulted in a final sample size of 6693 participants, with TEI ranging from 754–3293 kcal/day. Participants were 13–45 years old, with a median age of 28.0 years (IQR 23.0–31.0). Seventy-eight percent of participants identified as White, and 16% identified as Hispanic. Nearly 25% of the sample had public insurance, 16% had a high school education or less, and 13% had a household income less than the federal poverty level. Only 4% of the sample had a preexisting chronic medical condition such as pregestational diabetes or chronic hypertension. As for other indicators of metabolic health, the median pre-pregnancy BMI was 24.5 kg/m^2^ (22.0–28.9), and participants reported a median of 13.3 (7.0–23.2) metabolic equivalent (MET) minutes of physical activity per week. Study participants typically delivered at 39.6 weeks’ gestation (38.7–40.4) and the median birthweight was 3325 g (3000–3635) (Table 1).

The mean caloric intake for the sample was 1620 ± 541 kcal/day. On average, 51.3% of participants’ TEI was from UPF. As shown in Table 2, ultra-processed grains accounted for the highest percentage of UPFs consumed (13.2 ± 6.9% kcal/day), followed by entrée items (11.1 ± 6.7% kcal/day). Ultra-processed drinks and condiments accounted for nearly 10% of the average daily diet. UPF intake was inversely correlated with age (*r_s_* = −0.319, *p* < 0.001) and physical activity (*r_s_* = −0.196, *p* < 0.001). While there were statistically significant correlations with pre-pregnancy BMI (*r_s_* = 0.031, *p* = 0.01), gestational age at birth (*r_s_* = −0.088, *p* < 0.001), and birthweight (*r_s_* = −0.059, *p* < 0.001), these correlations were very weak.

UPF intake varied across nearly all demographic and baseline clinical characteristics (Table 1). Patients with higher UPF intake were more likely to be Black or African American, non-Hispanic, publicly insured, have less than a college education, use tobacco during pregnancy, and have a household income less than the federal poverty level (all *p*-values < 0.001). Mean UPF intake did not significantly differ between patients with and without pregestational diabetes or NICU admission status; however, UPF intake was higher in patients with chronic hypertension (*p* = 0.02) and those who had a vaginal delivery (*p* = 0.002).

Table 3 describes the associations between UPF intake and adverse pregnancy outcomes. Overall, 8% of the sample experienced preterm birth, 9% had SGA, 8% had LGA, 24% had HDP, and 5% had GDM. In the bivariate analysis, patients with the following pregnancy complications all had a significantly higher proportion of UPF within their diet compared to patients without these adverse outcomes: preterm birth (53.4 ± 12.9% kcal/day vs. 51.2 ± 12.7% kcal/day, *p* < 0.001), HDP (52.0 ± 12.6% kcal/day vs. 51.1 ± 12.7% kcal/day, *p* = 0.02), SGA (52.4 ± 13.1% kcal/day vs. 51.2 ± 12.7% kcal/day, *p* = 0.03), and fetal or neonatal demise (56.9 ± 11.3% kcal/day vs. 51.3 ± 12.7% kcal/day, *p* = 0.003). The mean proportion of UPF intake was not different by GDM or LGA incidence. After adjusting for education level, poverty status, insurance type, and chronic hypertension, increasing UPF consumption remained associated with preterm birth (AOR 1.11, 95% CI 1.02–1.21) and with HDP (AOR 1.05, 95% CI 1.001–1.11). Though UPF intake was associated with increased odds of SGA (OR 1.08, 95% CI 1.01–1.15) and fetal/neonatal demise (OR 1.42, 95% CI 1.13–1.78) in the unadjusted models, these associations were no longer significant after adjusting for confounders. Similar to our findings in the bivariate analysis, neither GDM nor LGA was associated with UPF intake in the logistic regressions.

To assess the effect of gastrointestinal conditions associated with malabsorption on our findings, we also conducted a sensitivity analysis, excluding all patients from the cohort with a documented condition associated with malabsorption. Of the 6693 participants meeting all inclusion criteria for the secondary analysis, 44 patients were identified to have a condition associated with malabsorption, including ulcerative colitis, Crohn’s disease, and celiac disease. After excluding these patients, UPF intake remained significantly associated with preterm birth (AOR 1.11, 95% CI 1.02–1.20) and HDP (AOR 1.05, 95% CI 1.001–1.11) (Supplementary Materials, Table S1).

## 4. Discussion

In this secondary analysis of a large, prospective observational pregnancy cohort, we evaluated associations between maternal characteristics and periconceptional UPF intake (defined as a percentage of TEI) and explored relationships between UPF intake and several adverse pregnancy outcomes. UPF intake differed significantly by several demographic and clinical characteristics. Results also showed that UPF intake was significantly higher among patients with pregnancies complicated by preterm birth, HDP, SGA, and fetal or neonatal demise. After adjusting for potential confounders, only the associations of UPF intake with preterm birth and HDP remained significant.

Similar to our findings, previously published studies investigating UPF intake during pregnancy using U.S.-based cohorts have also found that UPFs comprise around half of the maternal daily diet [10,11]. Additionally, our findings confirm that UPF intake during pregnancy varies significantly across maternal characteristics. A secondary analysis by Yisahak et al. demonstrated similar distributions of UPF intake, including less UPF consumption among older patients, patients with lower BMIs, and patients who identify as Asian or Asian American [19]. Interestingly, Yisahak et al. found that UPF intake was higher among patients with higher levels of physical activity, while our findings suggest the opposite.

Previous research has demonstrated mixed results regarding the relationship between UPF and preterm birth. For example, two meta-analyses of studies examining the relationship between UPF-rich diets and maternal–fetal outcomes found no significant association between UPF consumption and preterm birth [17,18]. Of note, both analyses used the NOVA Food Classification System, focusing on group 4 foods, similar to the approach we used in the current study. However, in studies that investigated general dietary patterns, rather than UPF consumption specifically, associations have been reported between different prenatal dietary patterns and preterm birth [17,18]. For example, a meta-analysis by Raghavan et al. concluded that dietary patterns aimed at reducing UPFs have limited but consistent evidence for reducing the risk of preterm birth [38]. Similarly, Gete et al. found that Western diets, known for consisting largely of UPFs, were associated with an increased risk of preterm birth, whereas the DASH diet, which emphasizes the intake of whole foods, was associated with decreased preterm birth risk [39]. Overall, to our knowledge, this is one of the first studies to use the NOVA classification system and demonstrate a significant relationship between UPF intake and increased risk of preterm birth.

Although the number of studies is limited, previous research consistently reports an association between UPF intake and increased risk of preeclampsia [17,18]. In a meta-analysis by Talebi et al., this relationship remained significant even in the subgroup analysis that included only studies using the NOVA food classification system to define UPFs [18]. While large epidemiological studies have been conducted in Scandinavian countries, to our knowledge, the current study is the largest analysis evaluating preeclampsia and UPF intake in a U.S.-based cohort [40,41,42].

In contrast, the relationship between UPF intake and SGA has not been well established. A systematic review by Paula et al. found several cohort studies that showed an increased risk of SGA with UPF consumption; however, the association was not significant in the meta-analysis [17]. In a different systematic review by Talebi et al., only one study was identified that found a significant relationship between UPF consumption and SGA; however, overall, they also concluded their meta-analysis found no significant association [18]. These findings, along with those of our study, suggest that additional research is needed to elucidate the relationship between UPF consumption and fetal growth.

Unlike other published studies, we did not find a significant relationship between UPF intake in early pregnancy and risk of GDM. Both meta-analyses described above found that UPF was significantly associated with an increased risk of GDM [17,18]. Notably, Talebi et al. demonstrated a dose-dependent association: a 100-g increase in UPF intake correlated with a 27% increase in GDM risk. The disproportionately low GDM incidence in the nuMoM2b cohort may have contributed to these findings, as well as to the non-significant association we found between UPF intake and risk of LGA infants, a common complication of GDM. Much like the meta-analysis performed by Paula et al., our study did not find an association between UPF intake and LGA incidence. However, some studies have shown a relationship between unhealthy dietary patterns in early pregnancy and LGA birthweight [43,44] and increased neonatal adiposity [11].

The association between fetal or neonatal demise and maternal UPF consumption has not been well explored. For example, in a prospective cohort study by Tamada et al., higher intake of ready-made and frozen meals was significantly associated with increased risk of stillbirth [45]. Notably, patients who consumed such foods at least 3–7 times per week demonstrated a twofold risk of stillbirth. However, there is a lack of additional data, beyond the Japan Environment and Children’s Study, looking specifically at the relationship between UPF consumption and stillbirth or early neonatal death.

Given the significant proportion of the maternal diet composed of UPFs, preconception or early-pregnancy education efforts are imperative to improve nutritional literacy. Specifically, pregnant patients and patients trying to conceive should be educated on how to identify UPFs and the potential risk of adverse pregnancy outcomes associated with high UPF intake. However, alongside prenatal education, large-scale efforts to improve awareness among reproductive-aged populations are also needed. In our study, we found that UPF intake was significantly higher among patients with preexisting metabolic conditions, such as chronic hypertension and obesity. One option explored in Europe is the placement of easy-to-read warnings or indicators on UPF product labels to help vulnerable groups identify them [46].

Beyond increasing general awareness, medical nutrition therapy (MNT) could potentially play an important role in at-risk patients. For example, MNT is a recommended first-line treatment for all patients diagnosed with GDM; however, it may be beneficial to explore integrating MNT for patients at high risk of other adverse pregnancy outcomes, given the findings of the current study and those previously published. Finally, patient education alone is likely insufficient to combat the rising popularity of UPFs in the average American diet. We found significant differences between UPF intake across sociodemographic characteristics, suggesting that UPF intake is highest among marginalized communities. Public policy initiatives should be implemented alongside clinical interventions to reduce food insecurity and optimize access to affordable, nutritious, minimally processed foods.

These findings add to the growing body of literature on UPF consumption and pregnancy-related outcomes and are especially timely given their rising popularity. While our study demonstrated associations between UPF intake and adverse pregnancy outcomes, the processes driving these pathways remain unclear. Proposed mechanisms include UPF-induced systemic inflammation [47], endocrine pathway disruption by non-nutritive additives associated with UPF [48], overall degradation of the diet causing deficiencies in key nutrients for pregnancy and development [48,49,50], and increased risk for cardiometabolic pregnancy complications [17,47]. Further, individuals with high UPF intake demonstrate gut microbiome dysregulation, including a predominance of inflammatory bacteria [47]. This may drive persistent inflammation that contributes to an increased risk of adverse pregnancy outcomes, and perhaps even lasting epigenetic modifications in the fetus [51,52]. Overall, additional research is needed to explore the biological pathways by which ultra-processed food components affect fetal development and maternal health to better understand the associations identified in the current study.

Another point of future research includes expanding the availability of validated tools that identify and characterize UPFs. Though the NOVA scale is a validated tool, it broadly classifies all ultra-processed foods into a single category, whereas many of these foods may vary in their nutritional content or preparation techniques [53]. Further, accurately classifying and quantifying UPF intake is challenging and requires robust dietary assessments that can be burdensome for participants. There are also limited instruments for specifically assessing and quantifying UPF intake, potentially leading to wide subjectivity and variability in how foods are categorized by the NOVA scale [54,55]. Research is needed to develop and validate such tools across diverse populations to better understand the relationship between UPFs and pregnancy outcomes.

One strength of this study is the use of the prospective, contemporary, multisite nuMoM2b cohort, which has a large, demographically diverse sample that mirrors the racial and ethnic diversity of the U.S. Additionally, by excluding multiparous women who may have previous adverse obstetric outcomes, it reduces the potential for confounding when investigating outcomes of the index pregnancy. However, generalizability to multiparas, who may be at higher risk of adverse pregnancy outcomes per their obstetric history, may be limited. In addition to the sample characteristics, the Modified Block FFQ provided a comprehensive assessment of periconceptional dietary intake, enabling more accurate estimation of UPF intake. Using FoodData Central to calculate the average caloric density of several food items also helped address variances across different brands or preparations of foods included in the FFQ. One limitation of the FFQ, however, is the potential for recall bias, as information was collected up to six months after the periconception window. There is also the potential for selection bias, as participants who did not complete the FFQ may have significantly different dietary patterns from those included in this analysis. Lastly, the incidence of GDM in this cohort was about half that in the U.S., limiting the potential generalizability of these findings and the conclusions we could draw regarding UPF intake and GDM incidence.

## 5. Conclusions

In this secondary analysis of nulliparous mothers, consumption of ultra-processed foods varied across demographic characteristics and was associated with increased risk of preterm birth and hypertensive disorders of pregnancy. These findings shed light on the importance of increasing access to affordable, nutritious foods and expanding patient education focusing on UPF intake during the periconceptional period.

## Figures and Tables

**Table 1:** Ultra-processed food intake across baseline demographic and clinical characteristics (*N* = 6693).

	*n*	Median (IQR)	Correlation (*r_s_*) with UPF Intake ^1^	*p*
**Age (y)**	6693	28.0 (23.0–31.0)	−0.319	<0.001
**Pre-pregnancy BMI (kg/m^2^)**	6598	24.5 (22.0–28.9)	0.031	0.01
**Physical activity per week, reported as total metabolic equivalents**	5962	13.3 (7.0–23.2)	−0.196	<0.001
**Gestational age at birth (weeks)**	6647	39.6 (38.7–40.4)	−0.088	<0.001
**Birthweight (grams)**	6623	3325 (3000–3635)	−0.059	<0.001
	***n***	***n* (%)**	**UPF intake as a percentage of daily intake** **(mean ± SD) ^2^**	***p***
**Ethnicity**	6693			<0.001
Not Hispanic or Latino		5603 (84)	51.6 ± 12.8	
Hispanic or Latino		1090 (16)	49.8 ± 12.5	
**Race**	6137			<0.001
American Indian/Alaska Native		7 (<1)	50.9 ± 9.3	
Asian		289 (5)	43.1 ± 12.3	
Black/African American		653 (11)	58.3 ± 12.4	
Native Hawaiian/Other Pacific Islander		23 (<1)	51.5 ± 9.6	
White		4797 (78)	51.0 ± 12.4	
More than one race		368 (6)	53.2 ± 12.9	
**Education**	6692			<0.001
High school education or less		1039 (16)	57.4 ± 13.1	
Greater than high school education		5653 (84)	50.2 ± 12.3	
**Insurance**	6661			<0.001
Public insurance		1574 (24)	54.9 ± 13.3	
Commercial insurance		5087 (76)	50.2 ± 12.3	
**Household income**	5682			<0.001
<100% of the federal poverty level		731 (13)	56.3 ± 12.9	
≥100% of the federal poverty level		4951 (87)	49.8 ± 12.3	
**Pregestational diabetes**	6642			0.88
No		6546 (99)	51.3 ± 12.7	
Yes		96 (1)	51.6 ± 14.6	
**Chronic hypertension**	6636			0.02
No		6481 (98)	51.3 ± 12.7	
Yes		155 (2)	53.7 ± 12.4	
**Tobacco exposure**	6689			<0.001
No		5616 (84)	51.0 ± 12.6	
Yes		1073 (16)	53.0 ± 13.3	
**Mode of delivery**	6642			0.002
Vaginal		4810 (72)	51.6 ± 12.8	
Cesarean		1832 (28)	50.6 ± 12.4	
**NICU admission**	6456			0.10
No		5557 (86)	51.3 ± 12.7	
Yes		899 (14)	52.0 ± 12.8	

UPF, ultra-processed food; SD, standard deviation; BMI, body mass index; IQR, interquartile range. ^1^ Spearman correlations were used to compare each continuous variable with UPF intake as a percentage of daily energy intake. ^2^ Two-sample *t*-tests were used to compare mean UPF intake as a percentage of daily energy intake.

**Table 2:** Daily ultra-processed food intake for all participants (*N* = 6693).

UPF Items from the FFQ	UPF Intake as a Percentage of Daily Food Intake (Mean ± SD)
**Ultra-processed grains:** Buns, tortillas (corn or flour), bread (white, wheat, low carb), cornbread, pancakes, cooked cereal, cold cereal (low carb, cheerios, total, fiber one, product 19, all bran, other fiber, sweetened, cornflakes), bagels, biscuits	13.2 ± 6.9%
**Processed meats and proteins:** Sausage, hamburger (with or without cheese), meat substitutes, hot dogs (including low fat), bologna (including low fat), fried chicken (with or without skin), fried fish (with or without skin), refried beans	5.2 ± 3.5%
**Entrees:** Soups, pizza, spaghetti (with or without meat), macaroni, other noodles, breakfast sandwiches	11.1 ± 6.7%
**Snacks:** Power bars, crackers, pretzels, salty snacks, breakfast bars (low carb, low fat), yogurt, fries	6.6 ± 4.7%
**Desserts:** Donuts, cookies, cakes, pudding, ice cream, pie, candy	6.2 ± 5.0%
**Condiments:** Salad dressing (including low carb and low fat), peanut butter, mustard, barbeque sauce, ketchup, mayo, salsa, jelly, chocolate syrup	3.9 ± 2.9%
**Drinks:** Diet shakes, HI-C, Kool-Aid, some juices, soft drinks	5.5 ± 7.4%
**Total UPF daily intake**	51.3 ± 12.7%

UPF, ultra-processed food; FFQ, Food Frequency Questionnaire; SD, standard deviation.

**Table 3:** Ultra-processed food consumption and risk of adverse pregnancy outcomes.

	*n*	*n* (%)	UPF Intake as a Percentage of Daily Intake (Mean ± SD)	*p*	OR ^1^ (95% CI), *p*	AOR ^2^ (95% CI), *p*
**Preterm birth**	6668			<0.001	1.15 (1.07–1.23), 0.002	1.11 (1.02–1.21), 0.02
No		6166 (92)	51.2 ± 12.7			
Yes		522 (8)	53.4 ± 12.9			
**HDP**	6636			0.02	1.06 (1.01–1.11), 0.02	1.05 (1.001–1.11), 0.04
No		5114 (77)	51.1 ± 12.7			
Yes		1522 (24)	52.0 ± 12.6			
**SGA**	6623			0.03	1.08 (1.01–1.15), 0.03	1.06 (0.98–1.14), 0.17
No		6024 (91)	51.2 ± 12.7			
Yes		599 (9)	52.4 ± 13.1			
**LGA**	6456			0.32	0.97 (0.90–1.04), 0.32	1.00 (0.92–1.08), 0.97
No		5936 (92)	51.4 ± 12.7			
Yes		518 (8)	50.8 ± 12.6			
**GDM**	6693			0.38	0.96 (0.88–1.05), 0.38	1.00 (0.90–1.10), 0.94
No		6382 (95)	51.4 ± 12.8			
Yes		311 (5)	50.7 ± 12.0			
**Fetal or neonatal demise ^3^**	6437			0.003	1.42 (1.13–1.78), 0.003	1.22 (0.91–1.62), 0.19
No		6391 (99)	51.3 ± 12.7			
Yes		46 (1)	56.9 ± 11.3			

UPF, ultra-processed food; SD, standard deviation; OR, odds ratio; CI, confidence interval; AOR, adjusted odds ratio; HDP, hypertensive disorders of pregnancy; SGA, small for gestational age; GDM, gestational diabetes. ^1^ Calculated per ten percent changes in UPF intake. ^2^ Adjusted for education level, poverty status, insurance type, and chronic hypertension. ^3^ Assessed up to 28 days of life.

## Data Availability

The original data presented in the study are openly available in the Eunice Kennedy Shriver National Institute of Child Health and Human Development (NICHD) Data and Specimen Hub (DASH) at https://dash.nichd.nih.gov/.

## Supplementary Materials

The following supporting information can be downloaded at: https://www.mdpi.com/article/10.3390/nu18040627/s1, Table S1: Ultra-processed food consumption and risk of adverse pregnancy outcomes after excluding patients with gastrointestinal conditions associated with malabsorption (*N* = 6634).

## Abbreviations

The following abbreviations are used in this manuscript:

UPF	Ultra-processed foods
APOs	Adverse pregnancy outcomes
nuMoM2b	Nulliparous Pregnancy Outcomes Study: Monitoring Mothers-to-Be
% kcal/day	Percentage of daily kilocalories
HDP	Hypertensive disorders of pregnancy
GDM	Gestational diabetes mellitus
SGA	Small for gestational age
LGA	Large for gestational age
OR	Odds ratio
AOR	Adjusted odds ratio
CI	Confidence interval
DASH	Data and Specimen Hub
FFQ	Food frequency questionnaire
FDC	FoodData Central
TEI	Total energy intake
SD	Standard deviation
IQR	Interquartile range
*r_s_*	Spearman correlation coefficients
MET	Metabolic equivalent task
BMI	Body mass index
MNT	Medical nutrition therapy
