# Supplementary material for: The Association Between Periconceptional Consumption of Ultra-Processed Food and the Incidence of Adverse Pregnancy Outcomes
Source: Nutrients. 2026 Feb 14;18(4):627. doi: 10.3390/nu18040627 (PMC12943490; doi:10.3390/nu18040627)
Supplement: Supplementary file 1 [file nutrients-18-00627-s001.zip › nutrients-4082927-supplementary.pdf]

Table S1. Ultra-processed food consumption and risk of adverse pregnancy outcomes after excluding with gastrointestinal patients conditions associated with malabsorption (N=6634)

	n	*n* (%)	UPF intake as a percentage of daily intake (mean ± SD)	*p*	OR[1] (95% CI), *p*	AOR[2] (95% CI), *p*
**Preterm birth**						
No	6632	6117 (92.2)	51.2 ± 12.7	<0.001	1.15 (1.07-1.23), <0.001	1.11 (1.02-1.20), 0.02
Yes		515 (7.8)	53.4 ± 12.9			
**HDP**						
No	6585	5073 (77)	51.1 ± 12.8	0.02	1.06 (1.01-1.11), 0.02	1.05 (1.001-1.11), 0.046
Yes		1512 (23)	52.0 ± 12.6			
**SGA**						
No	6575	5983 (91)	51.3 ± 12.7	0.03	1.08 (1.01-1.15), 0.04	1.06 (0.98-1.14), 0.19
Yes		592 (9)	52.4 ± 13.1			
**LGA**						
No	6409	5900 (92)	51.4 ± 12.7	0.48	0.98 (0.91-1.05), 0.48	1.01 (0.93-1.09), 0.81
Yes		509 (8)	51.0 ± 12.5			
**GDM**						
No	6634	6326 (95)	51.4 ± 12.8	0.35	0.96 (0.88-1.05), 0.35	0.99 (0.90-1.10), 0.89
Yes		308 (5)	50.7 ± 12.1			
**Fetal or neonatal demise[3]**	6391			0.002	1.43 (1.14-1.81), 0.002	1.23 (0.92-1.64), 0.17
No		6346 (99)	51.3 ± 12.7			
Yes		45 (1)	57.1 ± 11.3			

UPF, ultra-processed food; SD, standard deviation; OR, odds ratio; CI, confidence interval; AOR, adjusted odds ratio; HDP, hypertensive disorders of pregnancy; SGA, small for gestational age; GDM, gestational diabetes.

[1]Calculated per ten percent changes in UPF intake.

[2]Adjusted for education level, poverty status, insurance type, and chronic hypertension.

[3]Assessed up to 28 days of life.
